# ChatGPT’s Agreement with the Recommendations from the 18th St. Gallen International Consensus Conference on the Treatment of Early Breast Cancer

**DOI:** 10.3390/cancers16244163

**Published:** 2024-12-13

**Authors:** Naiba Nabieva, Sara Y. Brucker, Benjamin Gmeiner

**Affiliations:** 1 Department of Gynecology and Obstetrics, Friedrich-Alexander-Universität Erlangen-Nürnberg, 91054 Erlangen, Germany; 2 GynPraxis Dr. Ernst und Kolleginnen, 91054 Erlangen, Germany; 3 Department of Obstetrics and Gynecology, University of Tuebingen, 72076 Tuebingen, Germany; 4 Machine Learning and Data Analytics Lab, Department of Artificial Intelligence in Biomedical Engineering, Friedrich-Alexander Universität Erlangen-Nürnberg, 91052 Erlangen, Germany

**Keywords:** breast cancer, SGBCC, artificial intelligence, AI, LLM, ChatGPT

## Abstract

Introduction: Organizations like the European Society for Medical Oncology and the St. Gallen Oncology Conference panel regularly review the latest research data to align on common recommendations for the treatment of breast cancer patients. In the era of artificial intelligence (AI), the question arises whether AI can enhance scientific debates by providing potential recommendations for expert discussions. Methods: We focused on the St. Gallen International Breast Cancer Conference (SGBCC) in 2023, where 71 experts from 27 countries answered 127 questions across 17 topics related to early breast cancer. OpenAI’s ChatGPT version 4.0 was employed to respond to the same set of questions. We simulated response variability and mitigated potential memory effects using several question-rounds in new chat sessions. Results: ChatGPT answered 71 questions (55.91%) in accordance with the most common answer voted by the SGBCC panel and showed a moderate overall agreement. In these cases, AI voted with an average reliability of 98.31%, compared to the panel’s average majority of 65.39% for the most common answer. A very high agreement could be observed in questions on “Genetics”, “Pathology”, “Oligometastatic disease”, “Ductal carcinoma in situ” and “Well-being for breast cancer survivors”. A very low agreement was seen in the topics “BRCA associated”, “Adjuvant endocrine therapy”, “HER2 positive”, “Local/regional recurrence” and “Bone-modifying therapy”. Conclusions: Our study demonstrates that ChatGPT shows potential in the development of breast cancer treatment recommendations, particularly in areas where high agreement with expert panel responses was observed. However, significant improvements are necessary before AI can be considered a reliable tool to support human expertise.

## Simple Summary

Organizations like the European Society for Medical Oncology and the St. Gallen Oncology Conference panel regularly review the latest research data to align on common recommendations for the treatment of breast cancer patients. In the era of artificial intelligence (AI), the question arises whether AI can support expert discussions. To our knowledge, this is the first analysis to explore the potential role of ChatGPT in developing breast cancer treatment recommendations based on the St. Gallen International Breast Cancer Conference questionnaire, which consisted of 127 questions across 17 topics related to early breast cancer. ChatGPT answered 71 questions (55.91%) in accordance with the most common answer voted by the panel and showed a moderate overall agreement. Our study demonstrates that ChatGPT shows potential in the development of breast cancer treatment recommendations, particularly in certain topics where high agreement with expert panel responses was observed.

## 1. Introduction

Breast cancer is the most common cancer in women worldwide [1,2]. The rapid development of effective novel drugs over the past few decades has led to improved survival rates across all subtypes of breast cancer [2,3,4,5,6,7]. However, to support physicians in recommending the most appropriate treatment options to their patients, the quickly evolving research and therapy landscape needs to be continuously monitored, structured and consolidated.

Several international and national organizations, such as the European Society for Medical Oncology (ESMO), the American Society of Clinical Oncology (ASCO), the National Comprehensive Cancer Network (NCCN) and the St. Gallen Oncology Conference panel, provide clinical practice guidelines based on the latest research data [8,9,10,11,12,13,14,15,16,17,18]. These organizations review and discuss international literature to align on common recommendations for the diagnosis and treatment of breast cancer patients [19].

In the era of artificial intelligence (AI), the question arises whether AI can enhance and support scientific debates by accessing data pools and providing potential recommendations for expert discussions. ChatGPT-4 (Chatbot Generative Pre-trained Transformer-4), with its 175 billion parameters, is currently one of the most powerful large language models, excelling in analyzing complex (medical) literature with the promise of providing insightful treatment recommendations [20,21].

To evaluate the usability of ChatGPT for developing treatment guidelines, it is of interest to compare its responses to medical questions with those given by medical experts. As most of the associations that provide recommendations do not use a publicly available question/answer format, it is not possible to objectify the expert discussions to be able to directly compare their conclusions to AI’s statements. The St. Gallen Oncology Conference, however, publishes its recommendations for the diagnosis and treatment of early breast cancer based on expert responses to a range of questions, making this questionnaire appropriate for a direct comparison.

This study aims to assess the role of AI in developing treatment recommendations and its potential to support the creation of breast cancer guidelines.

## 2. Methods

### 2.1. St. Gallen International Consensus Conference

The St. Gallen International Breast Cancer Conference (SGBCC) takes place every two years in Vienna, Austria. The first three days typically focus on medical education on early breast cancer through review lectures, while the fourth day features an international expert discussion that is open to the registered SGBCC audience. The expert panel, mainly consisting of gynecologists, surgeons, radiologists and pathologists, answers questions on key topics relevant to the diagnosis and treatment of early breast cancer. The panelists also discuss selected questions and voting results during the session and within a comprehensive manuscript published post-conference to make recommendations for everyday clinical practice based on a majority vote [8].

### 2.2. SGBCC Panel and Questionnaire 2023

We focused on the most recent (18th) SGBCC in March 2023, where 71 panelists from 27 countries (Table 1) and different disciplines answered 127 questions on early breast cancer. The questions included Yes/No, True/False and multiple-choice questions. Apart from one question, panelists could abstain from voting. This comprehensive questionnaire provided a wide range of topics and question formats, making it an ideal tool to assess the performance of ChatGPT.

### 2.3. ChatGPT Survey

We employed ChatGPT-4 (version 4.0) to respond to the same 127 questions posed to the SGBCC experts. The model was trained on a dataset comprising publicly available and licensed data up to April 2023. The specific SGBCC recommendations discussed in this study were not publicly available at the time of ChatGPT-4′s training. As such, the AI’s responses reflect its ability to synthesize existing general medical knowledge rather than predict or replicate specific panel outcomes.

Data processing was done using Python (version 3.11.4), specifically employing the pandas library (version 2.1.1) to extract and organize the questions, options and expert answers. For each question, responses were generated using Open AI’s GPT-4 model, which was prompted via API (Application Programming Interface) to select one of the given options multiple times (*n* = 5) to simulate response variability. To mitigate potential memory effects and associated learning by ChatGPT, each of the five question rounds was conducted in a new chat session. This approach ensured that the AI’s responses were not influenced by previous interactions, maintaining the integrity of the data.

### 2.4. Statistical Analysis and Agreement Between ChatGPT and the SGBCC Panel

We evaluated the agreement between the responses generated by ChatGPT and those provided by the SGBCC expert panel using different statistical measures.

Overall Percent Agreement: This measure represents the percentage of questions where both ChatGPT and the panel provided the same most common answer. It is a straightforward and clinically relevant metric that reflects how often the AI’s responses match the experts’ majority votes. To determine the concordance between ChatGPT and the expert panel, we compared for each question the panel’s majority answer with the answer that ChatGPT provided most often out of the five question rounds. Thus, in cases where both ChatGPT and the panel gave the same answer most commonly, the result was seen as concordant; otherwise, it was considered discordant.

Additionally, we assessed ChatGPT’s reliability by evaluating how consistently it provided the same answer out of five rounds. This demonstrates the robustness and stability of ChatGPT’s responses.

Pearson Correlation Coefficient: To assess the degree of agreement between the expert panel and ChatGPT responses, we used the Pearson correlation coefficient (*r*). This statistical measure evaluates the linear correlation between two sets of data, in this case, the response distributions of ChatGPT and the expert panel. For each question, the distribution of responses was represented as percentages across the different answer categories. The Pearson correlation coefficient was then calculated between these two distributions to quantify their similarity.

The Pearson correlation coefficient ranges from −1 to 1, with values closer to 1 indicating stronger positive linear relationship, values closer to −1 indicating a negative relationship, and values near 0 suggesting no linear relationship. The interpretation of r values adheres to the following commonly accepted guidelines:

- Very high agreement: *r* = 0.90–1.00
- High agreement: *r* = 0.70–0.89
- Moderate agreement: *r* = 0.50–0.69
- Low agreement: *r* = 0.30–0.49
- Very low agreement: *r* < 0.30

Using the Pearson correlation coefficient provided a quantitative measure of how closely ChatGPT’s response patterns aligned with those of the expert panel, enabling an assessment of its potential utility in supporting breast cancer treatment recommendations.

## 3. Results

**Figure 1:**
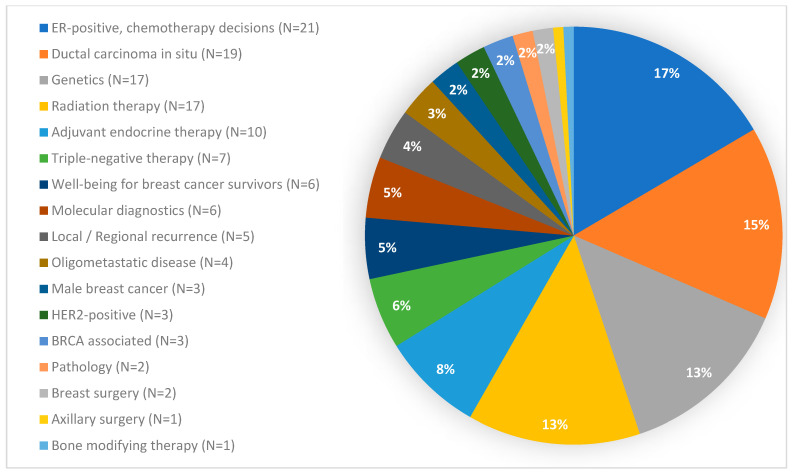
Questions per topic. BRCA: Breast Cancer gene; ER: estrogen receptor; HER2: human epidermal growth factor receptor 2.

The 127 questions posed to the SGBCC panel in 2023 covered 17 different topics and consisted of 52 Yes/No, 8 True/False and 67 multiple-choice questions, resulting in a distribution of 47% binary to 53% non-binary questions. With 21 questions, the topic “chemotherapy decisions in ER-positive disease” included the highest number of questions, while the topics “axillary surgery” and “bone modifying therapy” were represented with one question each (Figure 1).

ChatGPT answered 71 out of 127 (55.91%) questions in accordance with the most common answer voted by the SGBCC panel, resulting in a moderate overall agreement (*r* = 0.680). In these 71 questions, AI voted with an average reliability of 98.31%, i.e., out of five rounds per question, ChatGPT selected the most common answer in 98.31% of all cases. Out of a total of 127 questions, ChatGPT selected in 114 questions an answer with a reliability of at least 80%. The AI never abstained from voting (Table 2).

In those 71 questions where ChatGPT and the majority of the SGBCC panel chose the same answer, the panel voted with an average majority of 65.39% for the most common answer. Out of a total of 127 questions, 14 questions were answered by the experts with an average majority of at least 80%. The panelists abstained from voting with an average proportion of 11.02%. The average majority with which the panel voted was highest in questions with a very high agreement (70.69%) (Table 2).

Looking at the question type, ChatGPT answered 58.33% of the binary questions (i.e., Yes/No or True/False) and 53.73% of the non-binary questions (i.e., the multiple-choice ones) in accordance with the most common answer voted by the SGBCC panel (median *r* = 0.801 and 0.673, i.e., high and moderate agreement, respectively). With a median Pearson correlation coefficient of *r* = 0.931, the agreement between ChatGPT and panel responses was very high in True/False questions, but moderate in multiple choice (0.673) and Yes/No (0.663) questions (Table 3).

Regarding the topic, a very high agreement could be observed in questions on “Genetics”, “Pathology”, “Oligometastatic disease”, “Ductal carcinoma in situ”, and “Well-being for breast cancer survivors”. A very low agreement was seen in the topics “*BRCA* (BReast CAncer gene) associated”, “Adjuvant endocrine therapy”, “HER2 (human epidermal growth factor receptor 2) positive”, “Local/regional recurrence” and “Bone-modifying therapy” (Table 4).

## 4. Discussion

To our knowledge, this is the first analysis to explore the potential role of ChatGPT in developing breast cancer treatment recommendations based on the SGBCC questionnaire. Our results show that ChatGPT can provide support in certain areas, particularly where the agreement with expert panel responses is high. Topics such as “Genetics”, “Pathology”, “Oligometastatic disease”, “Ductal carcinoma in situ” and “Well-being for breast cancer survivors” showed very high agreement, while the model’s performance was less satisfactory in areas like “*BRCA* associated”, “Adjuvant endocrine therapy”, “HER2 positive”, “Local/regional recurrence” and “Bone-modifying therapy”. These discrepancies highlight the limitations of ChatGPT, which may stem from the complexity and specificity of medical knowledge required for certain topics.

It can be assumed that the high agreement observed in questions related to “well-being for breast cancer survivors” is due to the availability of substantial data on this topic, not only published in scientific literature but also in general media, which is accessible to both ChatGPT and the panel. Similarly, in questions on genetics, ChatGPT showed a very high level of agreement and answered 14 out of 17 questions in accordance with the panel, demonstrating a high level of knowledge in this area. While there were discrepancies in questions on genetic testing, ChatGPT achieved high to very high agreement scores on questions regarding risk-reducing mastectomy. In contrast, ChatGPT did not perform well in the area of adjuvant endocrine therapy (very low agreement with a median *r*= −0.012). A possible explanation is that this area is rapidly evolving, making it difficult for AI to assess and organize the most up-to-date data effectively [9,11,22,23]. Looking at the 21 questions on chemotherapy decisions in ER (estrogen receptor)-positive disease, in those 11 questions where ChatGPT did not agree with the panel’s majority votes, the panel also voted with an average majority of only 46.29%. In questions with a high to very high agreement, however, the average majority was 67.58%. Therefore, it can be concluded that ER-positive disease in general is an area where even human experts currently see challenges in making treatment recommendations. Regarding the topic “Radiation therapy”, most discrepancies between AI and panel were observed in questions on postmastectomy irradiation, where the experts, however, showed an average majority of 69.94%. Thus, ChatGPT seems to lack the required knowledge in this area (Supplementary Table S1).

At this point, it should be noted that version 4.0 of ChatGPT was trained on data up to April 2023, while the SGBCC took place in mid-March 2023. Therefore, the information necessary to answer the questions would theoretically have been available to a comparable extent to both the AI and the experts. Given OpenAI’s documentation of its training methodology and the timing of the SGBCC meeting, it is unlikely that ChatGPT-4 was trained on or influenced by the experts’ recommendations. Instead, areas of agreement likely stem from shared reliance on well-established scientific principles and treatment guidelines.

An interesting fact that could be observed is that ChatGPT answered the 127 questions with an average reliability of 96.22% and showed throughout a high reliability independent of the level of agreement with the panel. The experts, by comparison, reached only an average majority of 61.17%, which even decreased to 46.66% in questions with a low agreement with ChatGPT (Table 2). On the one hand, this can be interpreted as a favorable result, as AI does not randomly alternate between the answering options, but rather is consistent in the majority of the cases, suggesting a certain reliability. On the other hand, this data could also be interpreted as a limitation. Unlike human experts, who may consider a broader range of information and perspectives that result in diverse opinions, AI does not exhibit such variability in opinion.

Several analyses have been published on how ChatGPT can be used in the area of diagnosis and treatment of breast cancer. Roldan-Vasquez et al. compared the answers of ChatGPT with those of breast surgical oncologists to questions on breast cancer surgery and also found a good reliability, with an average reliability score of 3.98 out of 5.00 [24]. In a study on mammography recommendations in older women, ChatGPT answered 64% of the questions appropriately [25]. Patel et al. analyzed ChatGPT’s role in genetic counseling for gynecologic cancers and showed, similar to our results in genetic questions, that the chatbot is very well trained in this area, as it answered 33/40 (82.5%) of the questions correctly [26]. Another study compared ChatGPT to Bing AI in questions from the American Cancer Society’s recommended “Questions to Ask About Your Cancer” customized for all stages of breast, colon, lung and prostate cancer. In questions on breast cancer, ChatGPT did significantly better than Bing AI (score [scores from 1 = low to 5 = high for quality of information] of 4.1 vs. 2.9, *p* < 0.001) [27]. More complex approaches were chosen in studies where the investigators presented selected patient cases to ChatGPT and compared the recommendations of AI to those given by a tumor board [28,29,30,31]. Lukac et al., for instance, demonstrated in 10 patient cases how the chatbot provides recommendations on surgery, chemotherapy, radiotherapy and endocrine treatment. However, the performance was weak, achieving only 16.05% congruence with the tumor board, probably due to using the older version 3.5 [30]. Griewing et al., showed later that, in contrast to older versions, ChatGPT version 4.0 had the highest concordance with the tumor board [28,31]. Interestingly, contrary to our results, the authors observed full concordance for radiotherapy recommendations [28].

Our analysis has several limitations that should be considered when interpreting the results. First of all, some areas were represented by a very small number of questions, making it difficult to derive any conclusions for these topics. Furthermore, it is important to acknowledge that the reliability, with which we described ChatGPT’s assurance to provide a certain answer, naturally cannot be equated with the multiple number of panelists providing a response based on their personal knowledge, experience and opinion. Despite the fact that ChatGPT had been asked each question five times, the likelihood is high that it provided the same answer in most of the cases, so that the procedure is not comparable to asking five different experts. Another notable limitation of this study is that ChatGPT did not use the option to abstain from answering, unlike the human panelists. This slightly biases the results as, in contrast to AI, the panel “lost” this way some percentage points that probably could have led to a different majority decision and accordingly to a different agreement with ChatGPT for specific questions. Also, when comparing the panel’s answers with ChatGPT’s responses, we used the majority votes of the experts. However, the fact that the panel came to a majority vote does in general not mean that this specific answer is the only acceptable option. Thus, answers given by ChatGPT cannot be defined as correct or incorrect. Moreover, due to the complexity of each indication, differences in AI’s disease knowledge status, and the variable styles of treatment recommendation development across diverse associations, it is important to keep in mind that the results of our analysis cannot be directly translated to other areas.

The rapid evolution of large language models (LLMs) and the development of multi-modal AI systems may limit the long-term relevance of this study. As newer models with enhanced capabilities are introduced, the applicability of our findings could change. However, the methodology established in this study provides a foundation for future evaluations and comparisons as AI technology progresses.

In summary, the type of question does not appear to significantly affect ChatGPT’s performance, as agreement levels were similar for both binary and non-binary questions. However, ChatGPT demonstrates the ability to contribute to debates on topics in which it is well trained. This suggests that, in order to enhance scientific discussions, ChatGPT should be trained further, especially in those areas where it showed a weak performance. If trained well, its potential in co-developing recommendations and guidelines as an external “expert brain” could be of irreplaceable value. To learn more about the chatbot’s knowledge and understand its decision making, further investigations could involve asking ChatGPT to explain why it selected specific answers. Additionally, future analyses could benefit from comparing ChatGPT’s answers before and after the SGBCC, to determine any differences based on the timing of the survey.

## 5. Conclusions

Our study demonstrates that ChatGPT shows potential in the development of breast cancer treatment recommendations, particularly in areas where high agreement with expert panel responses was observed. However, significant improvements are necessary before AI can be considered a reliable tool to support human expertise. Future research should focus on enhancing the capabilities of AI and exploring its integration into the development process of clinical guidelines.

## Figures and Tables

**Table 1:** SGBCC panelists 2023 (ordered by surname).

Stephan Aebi (Switzerland)	Carsten Denkert (Germany)	Frederique Penault-Llorca (France)
Meteb Al-Foheidi (Kingdom of Saudi Arabia)	Gerd Fastner (Austria)	Martine Piccart (Belgium)
Fabrice André (France)	Florian Fitzal (Austria)	Lori Pierce (USA)
Mikola Anikusko (Ukraine)	Prudence Francis (Australia)	Philip Poortmans (Belgium)
Rajendra Badwe (India)	Heba Gamal (Egypt)	Meredith Regan (USA)
Andrea V. Barrio (USA)	Oreste Gentilini (Italy)	Jorge Reis-Filho (USA)
Carlos Barrios (Brazil)	Michael Gnant (Austria)	Isabella Rubio (Spain)
Jonas Bergh (Sweden)	William J. Gradishar (USA)	Hope Rugo (USA)
Hervé Bonnefoi (France)	Bahadir Gulluoglu (Turkey)	Emiel J. T. Rutgers (Netherlands)
Denisse Bretel Morales (Peru)	Nadia Harbeck (Germany)	Cristina Saura (Spain)
Sara Y. Brucker (Germany)	Jörg Heil (Germany)	Elzbieta Senkus (Poland)
Harold J. Burstein (USA)	Chiun-Sheng Huang (Taiwan)	Zhiming Shao (China)
Carlos Caldas (Great Britain)	Jens Huober (Switzerland)	Christian Singer (Austria)
David Cameron (Great Britain)	Zefei Jiang (China)	Beat Thürlimann (Switzerland)
Fatima Cardoso (Portugal)	Orit Kaidar-Person (Israel)	Masakazu Toi (Japan)
Maria Joao Cardoso (Portugal)	Marleen Kok (Netherlands)	Sara Tolaney (USA)
Lisa Carey (USA)	Eun-Sook Lee (Korea)	Nicholas Turner (Great Britain)
Steven Chia (Canada)	Sherene Loi (Australia)	Andrew Tutt (Great Britain)
Charlotte Coles (Great Britain)	Sibylle Loibl (Germany)	Marie-Jeanne Vrancken-Peeters (Netherlands)
Javier Cortes (Spain)	Miguel Martin (Spain)	Toru Watanabe (Japan)
Giuseppe Curigliano (Italy)	Icro Meattini (Italy)	Walter Weber (Switzerland)
Jana de Boniface (Sweden)	Kathy D. Miller (USA)	Hans Wildiers (Belgium)
Suzette Delaloge (France)	Monica Morrow (USA)	Binghe Xu (China)
Angela DeMichele (USA)	Ann Patridge (USA)	

**Table 2:** Comparison between ChatGPT and the SGBCC panel.

	ChatGPT	Panel
	N or %	N or %
**Agreement**		
Common responses	71 (55.91%)	
Overall agreement according to median Pearson correlation coefficient *r*	*r* = 0.680 (Moderate)	
Average reliability/majority in common answers (N = 71)	98.31%	65.39%
**Overall performance**		
Questions answered with average reliability/majority of >80%	118 (92.91%)	14 (11.02%)
Abstained from voting	0%	11.02%
Average reliability/majority in all questions (N = 127)	96.22%	61.17%
Average reliability/majority in questions with very high agreement (N = 54)	99.26%	70.69%
Average reliability/majority in questions with high agreement (N = 9)	95.56%	53.06%
Average reliability/majority in questions with moderate agreement (N = 8)	100%	44.78%
Average reliability/majority in questions with low agreement (N = 13)	86.15%	46.66%
Average reliability/majority in questions with very low agreement (N = 43)	94.88%	58.34%
**Topics of special interest**		
Average reliability/majority in questions on “Well-being” (N = 6)	100%	73.35%
Average reliability/majority in questions on “Genetics” (N = 17)	95.29%	60.80%
Average reliability/majority in questions on “DCIS” (N = 19)	97.89%	58.58%
Average reliability/majority in questions on “Radiation” (N = 17)	95.29%	63.08%
Average reliability/majority in questions on “Endocrine therapy” (N = 10)	88.00%	68.69%
Average reliability/majority in questions on “ER-positive, chemotherapy” (N = 21)	99.05%	57.44%

ER: estrogen receptor; DCIS: ductal carcinoma in situ.

**Table 3:** Agreement between ChatGPT and the SGBCC panel dependent on the type of question.

Question Type	Total		Agreement ChatGPT/Panel		Median Pearson Correlation Coefficient	Pearson Agreement
	N	%	N	%	*r*	
Binary (Yes/No and True/False)	60	47.24	35	58.33	0.801	High
Yes/No	52	40.94	30	57.69	0.663	Moderate
True/False	8	6.30	5	62.50	0.931	Very high
Non-binary (Multiple-choice)	67	52.76	36	53.73	0.673	Moderate

**Table 4:** Agreement between ChatGPT and the SGBCC panel dependent on the topic.

Topic (in the Order Displayed at SGBCC 2023)	Total		Agreement ChatGPT/Panel		Median Pearson Correlation Coefficient	Pearson Agreement
	N	%	N	%	*r*	
Well-being for breast cancer survivors	6	4.72	6	100	0.979	Very high
Pathology	2	1.57	2	100	0.922	Very high
Genetics	17	13.39	14	82.35	0.931	Very high
Ductal carcinoma in situ	19	14.96	10	52.63	0.945	Very high
Male breast cancer	3	2.36	2	66.67	0.673	Moderate
Radiation therapy	17	13.39	10	58.82	0.561	Moderate
Axillary surgery	1	0.79	1	100	0.793	High
Breast surgery	2	1.57	2	100	0.823	High
Local/Regional recurrence after breast cancer surgery/radiotherapy	5	3.94	0	0	−0.289	Very low
Adjuvant endocrine therapy	10	7.87	3	30.00	−0.012	Very low
ER-positive, chemotherapy decisions	21	16.54	11	52.38	0.772	High
Triple-negative therapy	7	5.51	4	57.14	0.812	High
HER2-positive	3	2.36	0	0	−0.020	Very low
*BRCA* associated	3	2.36	0	0	0.179	Very low
Bone modifying therapy	1	0.79	0	0	−0.344	Very low
Oligometastatic disease	4	3.15	4	100	0.975	Very high
Molecular diagnostics	6	4.72	2	33.33	0.365	Low
**In Total**	**127**	**100**	**71**	**55.91**	**0.680**	**Moderate**

BRCA: Breast Cancer gene; ER: estrogen receptor; HER2: human epidermal growth factor receptor 2.

## Data Availability

The original contributions presented in the study are included in the article/Supplementary Material, further inquiries can be directed to the corresponding author.

## Supplementary Materials

The following supporting information can be downloaded at: https://www.mdpi.com/article/10.3390/cancers16244163/s1. Table S1: Overview of the responses provided by the SGBCC panel and ChatGPT.
