# Supplementary material for: ChatGPT’s Agreement with the Recommendations from the 18th St. Gallen International Consensus Conference on the Treatment of Early Breast Cancer
Source: Cancers (Basel). 2024 Dec 13;16(24):4163. doi: 10.3390/cancers16244163 (PMC11674622; doi:10.3390/cancers16244163)
Supplement: Supplementary file 1 [file cancers-16-04163-s001.zip › cancers-3308803-supplementary.pdf]

Supplementary Table 1: Overview of the responses provided by the SGBCC panel and ChatGPT

#	Category	Question	Answer Options	**Expert Answers** [a) xx%, b) yy%, c) zz%, ...]	**ChatGPT Answers** [a) xx%, b) yy%, c) zz%, ...]	**Pearson correlation coefficient**
1	Well-being for Breast Cancer Survivors	In patients with BMI of more than 25, there is a specific diet that can lower the risk of breast cancer recurrence	['a) Yes', 'b) No', 'c) Abstain']	['25.0%', '73.21%', '1.79%']	['0.0%', '100.0%', '0.0%']	0.948
2	Well-being for Breast Cancer Survivors	Patients who are obese (BMI >30) should be encouraged to lose weight because	['a) General health prevention for conditions such as diabetes, hypertension, and arthritis', 'b) Because it affects breast cancer recurrence risk', 'c) a) and b)', 'd) Should not be approached in order to maintain her QoL and/or because\nsustained weight reduction is rarely achieved', 'e) Abstain']	['13.64%', '1.52%', '83.33%', '1.52%', '0.0%']	['0.0%', '0.0%', '100.0%', '0.0%', '0.0%']	0.988
3	Well-being for Breast Cancer Survivors	Acupuncture should be considered a standard treatment option for breast cancer survivors (and should be appropriately covered by insurance or national governments) to alleviate symptoms of arthralgias related to AI-based therapy and/or neuropathy related to chemotherapy	['a) Yes', 'b) No', 'c) Abstain']	['69.64%', '23.21%', '7.14%']	['100.0%', '0.0%', '0.0%']	0.969
4	Well-being for Breast Cancer Survivors	You are counseling a premenopausal woman who has a history of breast cancer on the safety of pregnancy after treatment. Currently, she is receiving ovarian function suppression and tamoxifen for a history of node-positive breast cancer, and is at the end of her second year of endocrine treatment. You discuss the recent data from the IBCSG /BIG /Alliance POSITIVE trial. Would you recommend interrupting endocrine therapy now if she has 4 or more involved axillary lymph nodes?	['a) Yes', 'b) No', 'c) Abstain']	['14.29%', '78.57%', '7.14%']	['0.0%', '100.0%', '0.0%']	0.996
5	Well-being for Breast Cancer Survivors	Would you recommend interrupting endocrine therapy now if she was 28 years old with a high likelihood of maintaining fertility for several more years?	['a) Yes', 'b) No', 'c) Abstain']	['16.67%', '77.78%', '5.56%']	['0.0%', '100.0%', '0.0%']	0.990
6	Well-being for Breast Cancer Survivors	Postmenopausal women on aromatase inhibitor therapy for ER+ breast cancers should not be offered intravaginal estrogens because of concerns over systemic estrogen exposure and impact of adjuvant treatment efficacy.	['a) True', 'b) False', 'c) Abstain']	['27.27%', '57.58%', '15.15%']	['0.0%', '100.0%', '0.0%']	0.961
7	Pathology	A 43 year old woman has undergone lumpectomy for a 1.6cm, node-negative, grade 3, triple negative breast cancer. The sTIL score is 75%. Which of the following would you recommend?	['a) Adjuvant chemotherapy', 'b) No adjuvant chemotherapy given the favorable prognosis', 'c) Abstain']	['87.93%', '8.62%', '3.45%']	['100.0%', '0.0%', '0.0%']	0.999
8	Pathology	Patient has undergone surgery for stage T1b TNBC, measuring 0.8cm. The tumor has a high TILs >50%. Which of the following would you recommend?	['a) Adjuvant chemotherapy', 'b) No adjuvant chemotherapy given the favorable prognosis', 'c) Abstain']	['58.18%', '34.55%', '7.27%']	['100.0%', '0.0%', '0.0%']	0.845
9	Genetics	Breast cancer patients should have access to genetic counselors	['a) Yes', 'b) No', 'c) Abstain']	['98.48%', '1.52%', '0.0%']	['100.0%', '0.0%', '0.0%']	1.000

10	Genetics	As part of routine care, all patients with breast cancer should ordinarily undergo germline genetic counseling and testing with a gene panel testing (8 or 9 genes, including high penetrance and intermediate penetrance genes such as BRCA1, BRCA2, PALB2, ATM, and CHEK2), regardless of family history or age.	['a) Yes', 'b) No', 'c) Abstain']	['37.88%', '62.12%', '0.0%']	['80.0%', '20.0%', '0.0%']	0.360
11	Genetics	As part of routine care, all patients with breast cancer should ordinarily undergo germline genetic counseling and may undergo testing for a limited panel of actionable, high penetrance genes, namely, BRCA1, BRCA2, and PALB2.	['a) Yes', 'b) No', 'c) Abstain']	['46.97%', '45.45%', '7.58%']	['100.0%', '0.0%', '0.0%']	0.529
12	Genetics	As part of routine care, all patients younger than 70 with breast cancer should ordinarily undergo germline genetic counseling and may undergo testing with a gene panel testing (8 or 9 genes, minimum) regardless of family history.	['a) Yes', 'b) No', 'c) Abstain']	['45.45%', '48.48%', '6.06%']	['100.0%', '0.0%', '0.0%']	0.444
13	Genetics	You are discussing risk-reduing mastectomy and intensified surveillance with a perimenopausal woman who has been diagnosed with early stage breast cancer. She has had genetic testing, and harbors a deleterious mutation. As part of a shared decision making process, she asks your opinion on management. Indicate if you would preferentially recommend either contralateral risk-recuding surgery, or intensive (mammogram and MRI-based) screening, or if you have no preference (either) for BRCA1, premenopausal.	['a) Surgery', 'b) Intensive screening', 'c) Abstain']	['66.67%', '13.63%', '19.7%']	['100.0%', '0.0%', '0.0%']	0.995
14	Genetics	You are discussing risk-reduing mastectomy and intensified surveillance with a perimenopausal woman who has been diagnosed with early stage breast cancer. She has had genetic testing, and harbors a deleterious mutation. As part of a shared decision making process, she asks your opinion on management. Indicate if you would preferentially recommend either contralateral risk-recuding surgery, or intensive (mammogram and MRI-based) screening, or if you have no preference (either) for BRCA1, postmenopausal	['a) Surgery', 'b) Intensive screening', 'c) Abstain']	['60.61%', '16.67%', '22.72%']	['100.0%', '0.0%', '0.0%']	0.992
15	Genetics	You are discussing risk-reduing mastectomy and intensified surveillance with a perimenopausal woman who has been diagnosed with early stage breast cancer. She has had genetic testing, and harbors a deleterious mutation. As part of a shared decision making process, she asks your opinion on management. Indicate if you would preferentially recommend either contralateral risk-recuding surgery, or intensive (mammogram and MRI-based) screening, or if you have no preference (either) for BRCA2, premenopausal	['a) Surgery', 'b) Intensive screening', 'c) Abstain']	['63.64%', '16.67%', '22.72%']	['100.0%', '0.0%', '0.0%']	0.993
16	Genetics	You are discussing risk-reduing mastectomy and intensified surveillance with a perimenopausal woman who has been diagnosed with early stage breast cancer. She has had genetic testing, and harbors a deleterious mutation. As part of a shared decision making process, she asks your opinion on management. Indicate if you would preferentially recommend either contralateral risk-recuding surgery, or intensive (mammogram and MRI-based) screening, or if you have no preference (either) for BRCA2, postmenopausal	['a) Surgery', 'b) Intensive screening', 'c) Abstain']	['42.42%', '31.82%', '25.76%']	['100.0%', '0.0%', '0.0%']	0.933

17	Genetics	You are discussing risk-reduing mastectomy and intensified surveillance with a perimenopausal woman who has been diagnosed with early stage breast cancer. She has had genetic testing, and harbors a deleterious mutation. As part of a shared decision making process, she asks your opinion on management. Indicate if you would preferentially recommend either contralateral risk-recuding surgery, or intensive (mammogram and MRI-based) screening, or if you have no preference (either) for PALB2, premenopausal	['a) Surgery', 'b) Intensive screening', 'c) Abstain']	['42.42%', '31.82%', '25.76%']	['100.0%', '0.0%', '0.0%']	0.933
18	Genetics	You are discussing risk-reduing mastectomy and intensified surveillance with a perimenopausal woman who has been diagnosed with early stage breast cancer. She has had genetic testing, and harbors a deleterious mutation. As part of a shared decision making process, she asks your opinion on management. Indicate if you would preferentially recommend either contralateral risk-recuding surgery, or intensive (mammogram and MRI-based) screening, or if you have no preference (either) for PALB2, postmenopausal	['a) Surgery', 'b) Intensive screening', 'c) Abstain']	['19.7%', '53.03%', '27.27%']	['100.0%', '0.0%', '0.0%']	-0.676
19	Genetics	You are discussing risk-reduing mastectomy and intensified surveillance with a perimenopausal woman who has been diagnosed with early stage breast cancer. She has had genetic testing, and harbors a deleterious mutation. As part of a shared decision making process, she asks your opinion on management. Indicate if you would preferentially recommend either contralateral risk-recuding surgery, or intensive (mammogram and MRI-based) screening, or if you have no preference (either) for ATM, premenopausal	['a) Surgery', 'b) Intensive screening', 'c) Abstain']	['9.09%', '72.73%', '18.18%']	['20.0%', '80.0%', '0.0%']	0.931
20	Genetics	You are discussing risk-reduing mastectomy and intensified surveillance with a perimenopausal woman who has been diagnosed with early stage breast cancer. She has had genetic testing, and harbors a deleterious mutation. As part of a shared decision making process, she asks your opinion on management. Indicate if you would preferentially recommend either contralateral risk-recuding surgery, or intensive (mammogram and MRI-based) screening, or if you have no preference (either) for ATM, postmenopausal	['a) Surgery', 'b) Intensive screening', 'c) Abstain']	['1.52%', '78.78%', '19.7%']	['0.0%', '60.0%', '40.0%']	0.884
21	Genetics	You are discussing risk-reduing mastectomy and intensified surveillance with a perimenopausal woman who has been diagnosed with early stage breast cancer. She has had genetic testing, and harbors a deleterious mutation. As part of a shared decision making process, she asks your opinion on management. Indicate if you would preferentially recommend either contralateral risk-recuding surgery, or intensive (mammogram and MRI-based) screening, or if you have no preference (either) for CHEK2, premenopausal	['a) Surgery', 'b) Intensive screening', 'c) Abstain']	['7.58%', '71.21%', '21.21%']	['0.0%', '100.0%', '0.0%']	0.979
22	Genetics	You are discussing risk-reduing mastectomy and intensified surveillance with a perimenopausal woman who has been diagnosed with early stage breast cancer. She has had genetic testing, and harbors a deleterious mutation. As part of a shared decision making process, she asks your opinion on management. Indicate if you would preferentially recommend either contralateral risk-recuding surgery, or intensive	['a) Surgery', 'b) Intensive screening', 'c) Abstain']	['1.52%', '78.78%', '19.7%']	['0.0%', '100.0%', '0.0%']	0.974

		(mammogram and MRI-based) screening, or if you have no preference (either) for CHEK2, postmenopausal				
23	Genetics	Patients with early breast cancer and pathological PALB2 mutations should be offered adjuvant PARP inhibitor therapy in accordance with the same criteria for adjuvant use in BRCA1/2 mutation carriers recognizing the absence of data supporting such a recommendation.	['a) Yes', 'b) No', 'c) Abstain']	['37.93%', '53.45%', '8.62%']	['0.0%', '100.0%', '0.0%']	0.765
24	Genetics	A patient has undergone hereditary genetic testing and has had tumor genomic testing. There is no hereditary mutation. However, there is a deleterious, somatic tumor mutation in BRCA1. Would you give adjuvant olaparib, if readily available?	['a) Yes', 'b) No', 'c) Abstain']	['48.28%', '46.55%', '5.17%']	['100.0%', '0.0%', '0.0%']	0.530
25	Genetics	25) A patient with a BRCA2 mutation has been diagnosed with ER positive HER2 positive breast cancer, with stage meeting OLYMPIA eligibility and will receive standard adjuvant therapies. Would you recommend adjuvant olaparib, if readily available	['a) Yes', 'b) No', 'c) Abstain']	['45.61%', '43.86%', '10.53%']	['100.0%', '0.0%', '0.0%']	0.538
26	Ductal Carcinoma in situ	The preferred radiation treatment plan and schedule as therapy for DCIS after breast conserving surgery in a postmenopausal woman with lower risk DCIS	['a) Whole breast, standard 25 fraction schedule', 'b) Whole breast, hypofractionated 15-16 fraction schedule', 'c) Partial breast, 5 fraction schedule', 'd) Any is reasonable', 'e) Abstain']	['0.0%', '33.85%', '20.0%', '23.08%', '23.08%']	['0.0%', '20.0%', '0.0%', '80.0%', '0.0%']	0.306
27	Ductal Carcinoma in situ	The preferred radiation treatment plan and schedule as therapy for DCIS after breast conserving surgery in a premenopausal woman with lower risk DCIS	['a) Whole breast, standard 25 fraction schedule', 'b) Whole breast, hypofractionated 15-16 fraction schedule', 'c) Partial breast, 5 fraction schedule', 'd) Any is reasonable', 'e) Abstain']	['0.0%', '50.0%', '4.69%', '20.31%', '25.0%']	['0.0%', '0.0%', '0.0%', '100.0%', '0.0%']	0.009
28	Ductal Carcinoma in situ	A healthy postmenopausal woman has undergone breast conserving surgery for DCIS. The DCIS is grade 1 to 2, without comedonecrosis, and spans less than 2cm. Should she receive radiation therapy?	['a) Yes', 'b) No', 'c) Abstain']	['53.85%', '29.23%', '16.92%']	['100.0%', '0.0%', '0.0%']	0.945
29	Ductal Carcinoma in situ	If the same patient will take endocrine therapy for ER+ DCIS, would you recommend radiation therapy?	['a) Yes', 'b) No', 'c) Abstain']	['40.0%', '43.08%', '16.92%']	['100.0%', '0.0%', '0.0%']	0.404
30	Ductal Carcinoma in situ	A healthy premenopausal woman has undergone breast conserving surgery for DCIS. The DCIS is grade 1 to 2, without comedonecrosis, and spans less than 2cm. Should she receive radiation therapy?	['a) Yes', 'b) No', 'c) Abstain']	['72.31%', '18.46%', '9.23%']	['100.0%', '0.0%', '0.0%']	0.991
31	Ductal Carcinoma in situ	If the same patient will take endocrine therapy for ER+ DCIS, would you recommend radiation therapy?	['a) Yes', 'b) No', 'c) Abstain']	['61.54%', '23.08%', '15.38%']	['100.0%', '0.0%', '0.0%']	0.988
32	Ductal Carcinoma in situ	A patient (age above 70) has undergone breast conserving surgery for DCIS with margins greater than 2mm. Does this patient require radiation therapy treatment?	['a) Yes', 'b) No', 'c) Abstain']	['24.62%', '52.3%', '23.08%']	['80.0%', '20.0%', '0.0%']	-0.232
33	Ductal Carcinoma in situ	A patient (age 50 to 65) has undergone breast conserving surgery for DCIS with margins greater than 2mm. Does this patient require radiation therapy treatment?	['a) Yes', 'b) No', 'c) Abstain']	['78.12%', '4.69%', '17.19%']	['100.0%', '0.0%', '0.0%']	0.987

34	Ductal Carcinoma in situ	A patient (age below 50) has undergone breast conserving surgery for DCIS with margins greater than 2mm. Does this patient require radiation therapy treatment?	['a) Yes', 'b) No', 'c) Abstain']	['79.69%', '1.56%', '18.75%']	['100.0%', '0.0%', '0.0%']	0.978
35	Ductal Carcinoma in situ	A patient (age above 70) with a DCIS smaller than 2cm has undergone breast conserving surgery for DCIS with margins greater than 2mm. Does this patient require radiation therapy treatment?	['a) Yes', 'b) No', 'c) Abstain']	['21.54%', '66.15%', '12.31%']	['0.0%', '100.0%', '0.0%']	0.987
36	Ductal Carcinoma in situ	A patient (age 50 to 65) with a DCIS smaller than 2cm has undergone breast conserving surgery for DCIS with margins greater than 2mm. Does this patient require radiation therapy treatment?	['a) Yes', 'b) No', 'c) Abstain']	['50.0%', '28.12%', '21.88%']	['100.0%', '0.0%', '0.0%']	0.977
37	Ductal Carcinoma in situ	A patient (age below 50) with a DCIS smaller than 2cm has undergone breast conserving surgery for DCIS with margins greater than 2mm. Does this patient require radiation therapy treatment?	['a) Yes', 'b) No', 'c) Abstain']	['60.94%', '17.18%', '21.88%']	['100.0%', '0.0%', '0.0%']	0.995
38	Ductal Carcinoma in situ	A patient (age above 70) with a DCIS without comedonecrosis has undergone breast conserving surgery for DCIS with margins greater than 2mm. Does this patient require radiation therapy treatment?	['a) Yes', 'b) No', 'c) Abstain']	['18.46%', '55.38%', '26.16%']	['0.0%', '100.0%', '0.0%']	0.980
39	Ductal Carcinoma in situ	A patient (age 50 to 65) with a DCIS without comedonecrosis has undergone breast conserving surgery for DCIS with margins greater than 2mm. Does this patient require radiation therapy treatment?	['a) Yes', 'b) No', 'c) Abstain']	['65.63%', '6.24%', '28.13%']	['0.0%', '100.0%', '0.0%']	-0.781
40	Ductal Carcinoma in situ	A patient (age below 50) with a DCIS without comedonecrosis has undergone breast conserving surgery for DCIS with margins greater than 2mm. Does this patient require radiation therapy treatment?	['a) Yes', 'b) No', 'c) Abstain']	['75.0%', '1.56%', '23.44%']	['0.0%', '100.0%', '0.0%']	-0.730
41	Ductal Carcinoma in situ	A patient (age above 70) with a DCIS smaller than 2cm and without comedonecrosis has undergone breast conserving surgery for DCIS with margins greater than 2mm. Does this patient require radiation therapy treatment?	['a) Yes', 'b) No', 'c) Abstain']	['15.62%', '73.44%', '10.94%']	['0.0%', '100.0%', '0.0%']	0.998
42	Ductal Carcinoma in situ	A patient (age 50 to 65) with a DCIS smaller than 2cm and without comedonecrosis has undergone breast conserving surgery for DCIS with margins greater than 2mm. Does this patient require radiation therapy treatment?	['a) Yes', 'b) No', 'c) Abstain']	['42.19%', '39.06%', '18.75%']	['0.0%', '100.0%', '0.0%']	0.390
43	Ductal Carcinoma in situ	A patient (age below 50) with a DCIS smaller than 2cm and without comedonecrosis has undergone breast conserving surgery for DCIS with margins greater than 2mm. Does this patient require radiation therapy treatment?	['a) Yes', 'b) No', 'c) Abstain']	['60.32%', '20.63%', '19.05%']	['0.0%', '100.0%', '0.0%']	-0.471
44	Ductal Carcinoma in situ	A healthy postmenopausal woman has undergone breast conserving surgery and radiation therapy for DCIS. She is discussing adjuvant endocrine therapy with you. Her principal goal is prevention of in-breast recurrence. She is concerned about familiar side effects, the modest benefits of endocrine treatment. Which of the following would you recommend?	['a) No endocrine treatment', 'b) Tamoxifen 5mg daily', 'c) Tamoxifen 20mg daily', 'd) Aromatase inhibitor', 'e) Either c) or d)', 'f) Abstain']	['28.57%', '39.29%', '16.07%', '1.79%', '8.93%', '5.36%']	['0.0%', '0.0%', '0.0%', '0.0%', '100.0%', '0.0%']	-0.260
45	Male Breast Cancer	The preferred local-regional therapy option for male breast cancer in non-BRCA1/2 mutation carriers is:	['a) Lumpectomy', 'b) Lumpectomy and radiation therapy', 'c) Conventional mastectomy', 'd) Nipple-sparing mastectomy', 'e) Abstain']	['0.0%', '36.36%', '41.82%', '12.73%', '9.09%']	['0.0%', '0.0%', '100.0%', '0.0%', '0.0%']	0.673
46	Male Breast Cancer	The preferred adjuvant endocrine therapy option for men with stage 1 (excluding minimal risk) ER+ Her2- breast cancer is:	['a) Tamoxifen', 'b) Aromatase inhibitor', 'c) GnRH agonist and	['80.0%', '4.62%', '6.15%', '3.08%', '6.15%']	['100.0%', '0.0%', '0.0%',	0.999

			tamoxifen', 'd) GnRH agonist and aromatase inhibitor', 'e) Abstain']		'0.0%', '0.0%']	
47	Male Breast Cancer	The preferred adjuvant endocrine therapy option for men with stage 3 ER+ Her2- breast cancer is:	['a) Tamoxifen', 'b) Aromatase inhibitor', 'c) GnRH agonist and tamoxifen', 'd) GnRH agonist and aromatase inhibitor', 'e) Abstain']	['50.77%', '4.62%', '9.23%', '27.69%', '7.69%']	['0.0%', '0.0%', '0.0%', '100.0%', '0.0%']	0.221
48	Radiation therapy	The preferred radiation schedule for chest wall irradiation, irrespective of nodal irradiation, after mastectomy:	['a) Moderate hypofractionation (15-16 fractions over 3 weeks)', 'b) Ultra-hypofractionation (5 fractions in 1 week)', 'c) Abstain']	['64.06%', '10.94%', '25.0%']	['100.0%', '0.0%', '0.0%']	0.967
49	Radiation therapy	The preferred radiation schedule for whole breast irradiation, irrespective of nodal irradiation, after breast conserving surgery for invasive cancer:	['a) Moderate hypofractionation (15-16 fractions over 3 weeks)', 'b) Ultra-hypofractionation (5 fractions in 1 week)', 'c) Abstain']	['60.94%', '15.63%', '23.44%']	['100.0%', '0.0%', '0.0%']	0.987
50	Radiation therapy	The preferred radiation schedule for whole breast irradiation after breast conserving surgery for DCIS:	['a) Moderate hypofractionation (15-16 fractions over 3 weeks)', 'b) Ultra-hypofractionation (5 fractions in 1 week)', 'c) Abstain']	['56.25%', '18.75%', '25.0%']	['100.0%', '0.0%', '0.0%']	0.988
51	Radiation therapy	For routine radiation therapy for early breast cancer, there is at present no clinical advantage to proton therapy over photon therapy.	['a) True', 'b) False', 'c) Abstain']	['73.44%', '3.13%', '23.44%']	['100.0%', '0.0%', '0.0%']	0.960
52	Radiation therapy	A boost dose to the primary tumor bed after breast conserving surgery is indicated in:	['a) Patients with at least 1 of the 4 following prognostic factors: high-grade cancers; extensive intraductal component (EIC); TNBC or HER2-\npositive subtypes; age <50 years', 'b) Patients who have at least 2 of the 4 following prognostic factors: high-grade cancers; extensive intraductal component (EIC); TNBC or\nHER2-positive subtypes; age <50 years', 'c) Patients who have at least 3 of the 4 following prognostic factors: high-grade cancers; extensive intraductal component (EIC); TNBC or HER2-positive subtypes; age <50 years', 'd) Patients who have all 4 following prognostic factors: high- grade cancers; extensive intraductal component (EIC); TNBC or HER2-positive subtypes; age <50 years', 'e) I have other criteria', 'f) Abstain']	['35.94%', '7.81%', '3.13%', '1.56%', '17.19%', '34.38%']	['60.0%', '40.0%', '0.0%', '0.0%', '0.0%', '0.0%']	0.394
53	Radiation therapy	A boost dose to the primary tumor bed after breast conserving surgery can be omitted if a pCR in the breast was obtained with preoperative systemic therapy.	['a) Yes', 'b) Yes in baseline clinical stage 1 or 2 but not stage 3', 'c) No', 'd) Abstain']	['12.7%', '23.81%', '25.4%', '38.1%']	['0.0%', '0.0%', '100.0%', '0.0%']	0.026

54	Radiation therapy	A healthy postmenopausal woman has been diagnosed with clinical stage 1 breast cancer. Imaging studies suggest no axillary disease. The tumor is 1.3cm in size, strongly ER and PR positive and low grade (1 of 3). SLN surgery is performed without evidence of cancer in the SLNs. The patient will be adherent with endocrine treatment. The age at which whole breast radiation therapy may appropriately be omitted is:	['a) >55', 'b) >60', 'c) >65', 'd) >70', 'e) >75', 'f) Offer radiation therapy regardless of age if life expectancy >15 years', 'g) Abstain']	['2.17%', '0.0%', '23.91%', '19.57%', '13.04%', '41.3%', '0.0%']	['20.0%', '60.0%', '0.0%', '20.0%', '0.0%', '0.0%', '0.0%']	-0.486
55	Radiation therapy	The most important clinical observation from the PRIME-II trial is that:	['a) Radiation lowers in-breast recurrence and therefore is effective', 'b) Radiation doas not alter survival, and therefore can be omitted', 'c) Abstain']	['63.46%', '26.92%', '9.62%']	['100.0%', '0.0%', '0.0%']	0.949
56	Radiation therapy	A postmenopausal woman has undergone mastectomy and axillary surgery for a T2 breast cancer with nodal involvement as below. The tumor is ER positive, and there is micrometastatic cancer in 1 lymph node. Would you recommend postmastectomy radiation therapy?	['a) Yes', 'b) No', 'c) Abstain']	['7.27%', '89.09%', '3.64%']	['100.0%', '0.0%', '0.0%']	-0.467
57	Radiation therapy	A postmenopausal woman has undergone mastectomy and axillary surgery for a T2 breast cancer with nodal involvement as below. The tumor is ER positive, and there is cancer in 1 lymph node. Would you recommend postmastectomy radiation therapy?	['a) Yes', 'b) No', 'c) Abstain']	['24.07%', '72.22%', '3.7%']	['100.0%', '0.0%', '0.0%']	-0.228
58	Radiation therapy	A postmenopausal woman has undergone mastectomy and axillary surgery for a T2 breast cancer with nodal involvement as below. The tumor is ER positive, and there is cancer in 2 lymph nodes. Would you recommend postmastectomy radiation therapy?	['a) Yes', 'b) No', 'c) Abstain']	['53.06%', '34.69%', '12.24%']	['100.0%', '0.0%', '0.0%']	0.836
59	Radiation therapy	A postmenopausal woman has undergone mastectomy and axillary surgery for a T2 breast cancer with nodal involvement as below. The tumor is ER positive, and there is cancer in 3 lymph nodes. Would you recommend postmastectomy radiation therapy?	['a) Yes', 'b) No', 'c) Abstain']	['94.12%', '3.92%', '1.96%']	['100.0%', '0.0%', '0.0%']	1.000
60	Radiation therapy	A postmenopausal woman has undergone mastectomy and axillary surgery for a T3 breast cancer with nodal involvement as below. The tumor is ER positive, and the lymph nodes are negative. Would you recommend postmastectomy radiation therapy?	['a) Yes', 'b) No', 'c) Abstain']	['49.12%', '45.61%', '5.26%']	['100.0%', '0.0%', '0.0%']	0.561
61	Radiation therapy	A postmenopausal woman has undergone mastectomy and axillary surgery for a T2 breast cancer with nodal involvement as below. The tumor is HER2 positive, and there is micrometastatic cancer in 1 lymph node. Would you recommend postmastectomy radiation therapy?	['a) Yes', 'b) No', 'c) Abstain']	['19.23%', '76.92%', '3.85%']	['100.0%', '0.0%', '0.0%']	-0.317
62	Radiation therapy	A postmenopausal woman has undergone mastectomy and axillary surgery for a T2 breast cancer with nodal involvement as below. The tumor is HER2 positive, and there is cancer in 1 lymph node. Would you recommend postmastectomy radiation therapy?	['a) Yes', 'b) No', 'c) Abstain']	['38.0%', '60.0%', '2.0%']	['100.0%', '0.0%', '0.0%']	0.138

63	Radiation therapy	A postmenopausal woman has undergone mastectomy and axillary surgery for a T2 breast cancer with nodal involvement as below. The tumor is TNBC, and there is micrometastatic cancer in 1 lymph node. Would you recommend postmastectomy radiation therapy?	['a) Yes', 'b) No', 'c) Abstain']	['22.92%', '70.83%', '6.25%']	['100.0%', '0.0%', '0.0%']	-0.269
64	Radiation therapy	A patient has been found to have a heterozygous, deleterious mutation in the ATM gene. Is this a contraindication to radiation therapy after breast conserving surgery?	['a) Yes', 'b) No', 'c) Abstain']	['20.75%', '73.58%', '5.66%']	['0.0%', '100.0%', '0.0%']	0.977
65	Axillary Surgery	A patient has undergone neoadjuvant treatment with AC/T chemotherapy, with a significant clinical response. At surgery, there is residual disease in the axilla. The tumor is triple-negative. For each of these scenarios, would you recommend completion axillary dissection or axillary radiation? Nodal burden: Macrometastasis in 1 of 3 SLN.	['a) Treatment choice: AxLND', 'b) Treatment choice: AxRT', 'c) Both', 'd) Neither', 'e) Abstain']	['46.94%', '20.41%', '28.57%', '2.04%', '2.04%']	['100.0%', '0.0%', '0.0%', '0.0%', '0.0%']	0.793
66	Breast surgery	In a postmenopausal patient with ER+/Her2- clinically node-negative breast cancer and two ipsilateral breast cancers in two neighboring quadrants that would be amenable two double tumorextomy with two separate incisions standard of care is:	['a) Mastectomy without reconstruction', 'b) Mastectomy with reconstruction', 'c) Double tumorectomy', 'd) Abstain']	['0.0%', '13.64%', '68.18%', '18.18%']	['0.0%', '0.0%', '100.0%', '0.0%']	0.966
67	Breast surgery	In a postmenopausal patient with ER+/Her2- clinically node-negative breast cancer and two ipsilateral breast cancers in two neighboring quadrants that would be amenable two double tumorextomy with two separate incisions standard of surgical care is:	['a) Mastectomy without reconstruction', 'b) Mastectomy with reconstruction', 'c) Double tumorectomy', 'd) Abstain']	['1.52%', '28.79%', '42.42%', '27.27%']	['0.0%', '0.0%', '100.0%', '0.0%']	0.680
68	Local-Regional Recurrence After Bcs/Rt	A 63 year old woman was treated 9 years ago for a stage 2, node-negative, breast cancer with lumpectomy and radiation therapy, as well as adjuvant systemic treatment. Now she has had ipsilateral tumor recurrence. No > grade 1 or only very localized grade 2 side effects at the level of the skin and/or the soft tissues are present. The tumor is ER positive and HER2 negative. Staging scans including axilla are negative. The lesion is <2cm in size, 3cm from nipple, and would be amenable to breast conserving surgery with acceptable aesthetic results. You would recommend:	['a) Mastectomy', 'b) Breast conserving surgery', 'c) Breast conserving surgery and irradiation', 'd) Abstain']	['25.45%', '14.55%', '58.18%', '1.82%']	['0.0%', '100.0%', '0.0%', '0.0%']	-0.289
69	Local-Regional Recurrence After Bcs/Rt	A 63 year old woman was treated 3 years ago for a stage 2, node-negative, breast cancer with lumpectomy and radiation therapy, as well as adjuvant systemic treatment, endocrine therapy stopped 2 years ago. Now she has had ipsilateral tumor recurrence. No > grade 1 or only very localized grade 2 side effects at the level of the skin and/or the soft tissues are present. The tumor is ER positive and HER2 negative. Staging scans are negative. The lesion is <2cm in size, 3cm from nipple, and would be amenable to breast conserving surgery with acceptable aesthetic results. You would recommend:	['a) Mastectomy', 'b) Breast conserving surgery', 'c) Breast conserving surgery and irradiation', 'd) Abstain']	['74.07%', '5.56%', '18.52%', '1.85%']	['0.0%', '100.0%', '0.0%', '0.0%']	-0.387
70	Local-Regional Recurrence After Bcs/Rt	A patient has developed isolated local regional recurrence while on adjuvant aromatase inhibitor therapy. The recurrence is fully exised and receives definitive local therapy. The preferred ongoing endocrine treatment is:	['a) None', 'b) Switch from NS-AI to exemestane, or vice-versa', 'c) Ongoing AI with CDK4/6i', 'd) Switch to fulvestrant', 'e) Swith to fulvestrant and CDK4/6i', 'f) Switch to tamoxifen', 'g) Switch to tamoxifen and CDK4/6i', 'h) Abstain']	['1.82%', '16.36%', '5.45%', '12.73%', '20.0%', '23.64%', '9.09%', '10.91%']	['0.0%', '0.0%', '100.0%', '0.0%', '0.0%', '0.0%', '0.0%', '0.0%']	-0.390

71	Local-Regional Recurrence After Bcs/Rt	A patient has developed isolated local regional recurrence while on adjuvant aromatase inhibitor therapy. The recurrence is fully exised and receives definitive local therapy. The tumor is strongly ER positive and HER2 negative. When originally diagnosed, she had not received adjuvant chemotherapy. Would you recommend adjuvant chemotherapy?	['a) Yes', 'b) No', 'c) Abstain']	['27.78%', '62.96%', '9.26%']	['100.0%', '0.0%', '0.0%']	-0.176
72	Local-Regional Recurrence After Bcs/Rt	A patient has develpoed isolated local regional recurrence 4 years after initial diagnosis while on adjuvant aromatase inhibitor therapy. The tumor is strongly ER positive and Her2 negative. A staging work up is negative. The recurrence is fully exised and receives definitive local therapy. When originally diagnosed, she had not received adjuvant chemotherapy. Would you recommend genomic signature testing to decide whether to receive chemotherapy?	['a) Yes, to determine suitability fo chemotherapy', 'b) No, because will recommend chemotherapy regardless to given resistance to ET', 'c) No, because would not recommend chemotherapy', 'd) No, because will make clinical decision on other factors (grade, Ki67, PR status, age)', 'e) Depends on size of recurrent lesion', 'f) Abstain']	['9.09%', '14.55%', '16.36%', '52.73%', '1.82%', '5.45%']	['60.0%', '0.0%', '0.0%', '40.0%', '0.0%', '0.0%']	0.402
73	Adjuvant Endocrine Therapy	For tumors that are ER low-positive (less than 10%), the appropriate threshold for recommending adjuvant ET is (multiple answer possible):	['a) Any', 'b) 1%', 'c) 2%', 'd) 5%', 'e) 9%', 'f) >10%']	['18.03%', '57.38%', '59.02%', '67.21%', '85.25%', '100.0%']	['60.0%', '0.0%', '0.0%', '0.0%', '40.0%', '0.0%']	-0.524
74	Adjuvant Endocrine Therapy	Consider a patient with ER positive, HER2 negative breast cancer. The appropriate duration of endocrine therapy for someone with stage 1 disease is:	['d) 5 years', 'e) 7-8 years', 'f) 10 years', 'g) indefinite', 'h) no adjuvant ET', 'i) Abstain']	['88.24%', '9.8%', '1.96%', '0.0%', '0.0%', '0.0%']	['0.0%', '0.0%', '0.0%', '100.0%', '0.0%', '0.0%']	-0.232
75	Adjuvant Endocrine Therapy	Consider a patient with ER positive, HER2 negative breast cancer. The appropriate duration of endocrine therapy for someone with stage 2, node negative disease is:	['a) 5 years', 'b) 7-8 years', 'c) 10 years', 'd) indefinite', 'e) depending on further biological factors', 'f) Abstain']	['44.9%', '36.73%', '4.08%', '0.0%', '12.24%', '2.04%']	['40.0%', '0.0%', '60.0%', '0.0%', '0.0%', '0.0%']	0.146
76	Adjuvant Endocrine Therapy	Consider a patient with ER positive, HER2 negative breast cancer. The appropriate duration of endocrine therapy for someone with stage 2, node positive disease is:	['a) 5 years', 'b) 7-8 years', 'c) 10 years', 'd) indefinite', 'e) Abstain']	['8.0%', '68.0%', '24.0%', '0.0%', '0.0%']	['0.0%', '0.0%', '100.0%', '0.0%', '0.0%']	0.078
77	Adjuvant Endocrine Therapy	Consider a patient with ER positive, HER2 negative breast cancer. The appropriate duration of endocrine therapy for someone with stage 3 disease is:	['a) 5 years', 'b) 7-8 years', 'c) 10 years', 'd) indefinite', 'e) Abstain']	['3.92%', '25.49%', '68.63%', '1.96%', '0.0%']	['0.0%', '0.0%', '100.0%', '0.0%', '0.0%']	0.935
78	Adjuvant Endocrine Therapy	For a patient with stage 2, ER positive, HER2 negative breast cancer, the duration of endocrine therapy should be based on established risk	['a) Yes', 'b) No', 'c) Abstain']	['96.97%', '1.52%', '1.52%']	['100.0%', '0.0%', '0.0%']	1.000

		factors such as stage and grade, treatment tolerability, and patient preferences.				
79	Adjuvant Endocrine Therapy	For a patient with stage 2, ER positive, HER2 negative breast cancer, a genomic assay should be used, if readily available, to determine the duration of ET.	['a) Yes', 'b) No', 'c) Abstain']	['30.3%', '60.61%', '9.09%']	['100.0%', '0.0%', '0.0%']	-0.102
80	Adjuvant Endocrine Therapy	Adjuvant abemaciclib should be recommended based on tumor stage and histology, irrespective of Ki67 expression.	['a) True', 'b) False', 'c) Abstain']	['77.27%', '13.64%', '9.09%']	['0.0%', '100.0%', '0.0%']	-0.447
81	Adjuvant Endocrine Therapy	A patient with ER positive, grade 2 breast cancer has undergone surgery and SLN biopsy for 2.3cm tumor, with a macrometastasis affecting 1 of 1 SLN. She will get chemotherapy and endocrine therapy. Which of the following would you recommend?	['a) No further treatment', 'b) Axillary dissection to see if additional LN are positive to inform choice of abemaciclib', 'c) Adjuvant Abemaciclib', 'd) Abstain']	['44.44%', '35.56%', '6.67%', '13.33%']	['0.0%', '40.0%', '60.0%', '0.0%']	-0.420
82	Adjuvant Endocrine Therapy	You are discussing neoadjuvant endocrine therapy with a 70 year old postmenopausal woman who has a clinical T3N1 breast cancer that is grade 2, ER and PR positive, and HER2 negative. The tumor has a low-risk genomic signature or otherwise low-risk features. She would prefer BCS. You suggest what likely duration of neoadjuvant endocrine treatment prior to surgery.	['a) Approximately 3 months', 'b) Approximately 6 months', 'c) Approximately 12 months', 'd) Until maximum response is achieved', 'e) Until she is a candidate for BCS', 'f) Proceed to MRM as it is unlikely she will be a candidate for BCS', 'g) Abstain']	['1.52%', '37.88%', '10.61%', '34.85%', '9.09%', '1.52%', '4.55%']	['0.0%', '100.0%', '0.0%', '0.0%', '0.0%', '0.0%', '0.0%']	0.671
83	ER positive, chemotherapy decisions	Giving a short course of endocrine therapy (2-4 weeks) before surgery, and monitor ist effect on Ki67, can provide valuable information for waiving chemotherapy	['a) True', 'b) False', 'c) Abstain']	['69.7%', '16.67%', '13.64%']	['100.0%', '0.0%', '0.0%']	0.999
84	ER positive, chemotherapy decisions	Based on the subset data from MINDACT, TAILORx, and RxPonder, women age 50 or less, who are premenopausal, with ER positive stage 1 or 2 breast cancer do not need tumor genomic signature profiling because they benefit from chemotherapiy	['a) True', 'b) False', 'c) Abstain']	['12.77%', '76.6%', '10.64%']	['0.0%', '100.0%', '0.0%']	1.000
85	ER positive, chemotherapy decisions	A 47 year old premenopausal woman has been diagnosed with a screen detected, ER positive, PR positive, Her2 negative breast cancer. The tumor is 1.6cm, grade 2, and node-negative. The recurrence score is 21. You would recommend:	['a) Tamoxifen', 'b) OFS and Tamoxifen', 'c) OFS and AI', 'd) Chemotherapy then ET', 'e) Abstain']	['28.89%', '22.22%', '17.78%', '24.44%', '6.67%']	['0.0%', '0.0%', '0.0%', '100.0%', '0.0%']	0.293
86	ER positive, chemotherapy decisions	A 47 year old premenopausal woman has been diagnosed with a screen detected, ER positive, PR positive, Her2 negative breast cancer. The tumor is 1.6cm, grade 2, and node-positive, affecting 1 of 3 SLN. The recurrence score is 21. You would recommend:	['a) Tamoxifen', 'b) OFS and Tamoxifen', 'c) OFS and AI', 'd) Chemotherapy then ET', 'e) Abstain']	['5.56%', '16.67%', '20.37%', '57.41%', '0.0%']	['0.0%', '0.0%', '0.0%', '100.0%', '0.0%']	0.931
87	ER positive, chemotherapy decisions	A 47 year old premenopausal woman has been diagnosed with a screen detected, ER positive, PR positive, Her2 negative breast cancer. The tumor is 1.6cm, grade 2, and node-negative. The recurrence score is 17. You would recommend:	['a) Tamoxifen', 'b) OFS and Tamoxifen', 'c) OFS and AI', 'd) Chemotherapy then ET', 'e) Abstain']	['46.15%', '17.31%', '26.92%', '3.85%', '5.77%']	['100.0%', '0.0%', '0.0%', '0.0%', '0.0%']	0.843
88	ER positive, chemotherapy decisions	A 47 year old premenopausal woman has been diagnosed with a screen detected, ER positive, PR positive, Her2 negative breast cancer. The tumor is 1.6cm, grade 2, and node-positive, affecting 1 of 3 SLN. The recurrence score is 17. You would recommend:	['a) Tamoxifen', 'b) OFS and Tamoxifen', 'c) OFS and AI', 'd) Chemotherapy then ET', 'e) Abstain']	['6.0%', '14.0%', '32.0%', '44.0%', '4.0%']	['0.0%', '0.0%', '0.0%',	0.772

					'100.0%', '0.0%']	
89	ER positive, chemotherapy decisions	A 47 year old premenopausal woman has been diagnosed with a screen detected, ER positive, PR positive, Her2 negative breast cancer. The tumor is 1.6cm, grade 2, and node-negative. The recurrence score is 11. You would recommend:	['a) Tamoxifen', 'b) OFS and Tamoxifen', 'c) OFS and AI', 'd) Chemotherapy then ET', 'e) Abstain']	['92.16%', '1.96%', '3.92%', '0.0%', '1.96%']	['100.0%', '0.0%', '0.0%', '0.0%', '0.0%']	0.999
90	ER positive, chemotherapy decisions	A 47 year old premenopausal woman has been diagnosed with a screen detected, ER positive, PR positive, Her2 negative breast cancer. The tumor is 1.6cm, grade 2, and node-positive, affecting 1 of 3 SLN. The recurrence score is 11. You would recommend:	['a) Tamoxifen', 'b) OFS and Tamoxifen', 'c) OFS and AI', 'd) Chemotherapy then ET', 'e) Abstain']	['17.65%', '23.53%', '29.41%', '29.41%', '0.0%']	['20.0%', '0.0%', '0.0%', '80.0%', '0.0%']	0.418
91	ER positive, chemotherapy decisions	A 34 year old premenopausal woman has been diagnosed with a palpable, ER positive, PR positive, Her2 negative breast cancer. The tumor is 1.6cm, grade 2, and node- negative. The recurrence score is 21. You would recommend:	['a) Tamoxifen', 'b) OFS and Tamoxifen', 'c) OFS and AI', 'd) Chemotherapy then ET', 'e) Abstain']	['3.92%', '7.84%', '25.49%', '60.78%', '1.96%']	['0.0%', '0.0%', '0.0%', '100.0%', '0.0%']	0.926
92	ER positive, chemotherapy decisions	A 34 year old premenopausal woman has been diagnosed with a palpable, ER positive, PR positive, Her2 negative breast cancer. The tumor is 1.6cm, grade 2, and node- positive, affecting 1 of 3 SLN. The recurrence score is 21. You would recommend:	['a) Tamoxifen', 'b) OFS and Tamoxifen', 'c) OFS and AI', 'd) Chemotherapy then ET', 'e) Abstain']	['0.0%', '2.0%', '6.0%', '90.0%', '2.0%']	['0.0%', '0.0%', '0.0%', '100.0%', '0.0%']	0.999
93	ER positive, chemotherapy decisions	A 34 year old premenopausal woman has been diagnosed with a palpable, ER positive, PR positive, Her2 negative breast cancer. The tumor is 1.6cm, grade 2, and node- negative. The recurrence score is 12. You would recommend:	['a) Tamoxifen', 'b) OFS and Tamoxifen', 'c) OFS and AI', 'd) Chemotherapy then ET', 'e) Abstain']	['29.17%', '25.0%', '31.25%', '14.58%', '0.0%']	['100.0%', '0.0%', '0.0%', '0.0%', '0.0%']	0.398
94	ER positive, chemotherapy decisions	A 57 year old woman has been diagnosed with a screen-detected breast cancer. She undergoes breast conserving surgery and SNB. The tumor is 0.7cm, grade 2, ER positive, PR low-positive, and HER2 negative, and node-negative. Would you order a genomic signature assay?	['a) Yes', 'b) No', 'c) Abstain']	['24.24%', '74.24%', '1.52%']	['100.0%', '0.0%', '0.0%']	-0.212
95	ER positive, chemotherapy decisions	A 57 year old woman has been diagnosed with a screen-detected breast cancer. She undergoes breast conserving surgery and SNB. The tumor is 1.7cm, grade 2, ER positive, PR low-positive, and HER2 negative, and node-negative. She is willing to take chemotherapy if there is some benefit. The recurrence score is 26. Would you give chemotherapy?	['a) Yes', 'b) No', 'c) Abstain']	['78.46%', '15.38%', '6.15%']	['100.0%', '0.0%', '0.0%']	0.993
96	ER positive, chemotherapy decisions	A 57 year old woman has been diagnosed with a screen-detected breast cancer. She undergoes breast conserving surgery and SNB. The tumor is 0.7cm, grade 2, ER positive, PR low-positive, and HER2 negative, and node-negative. A MammaPrint assay was sent and returned high risk. Would you give chemotherapy?	['a) Yes', 'b) No', 'c) Abstain']	['26.53%', '69.39%', '4.08%']	['100.0%', '0.0%', '0.0%']	-0.178
97	ER positive, chemotherapy decisions	A 61 year old woman presented with a clinically detected breast cancer and palpable axillary LN. Biopsy confirmed an invasive ductal carcinoma, grade 1, that was strongly ER positive, strongly PR positive, HER2 negative cancer, and had a Ki67 below 15%. She underwent mastectomy and axillary node dissection. The primary tumor was 5.1cm	['a) 2', 'b) 3', 'c) 4', 'd) 8', 'e) 10 or more', 'f) I would never give chemotherapy in a case like this.', 'g) Abstain']	['6.15%', '13.85%', '61.54%', '1.54%', '0.0%', '9.23%', '7.69%']	['0.0%', '100.0%', '0.0%', '0.0%', '0.0%',	-0.009

		(T3). The recurrence score was 10. What number of positive axillary lymph nodes would prompt you to recommend chemotherapy?			'0.0%', '0.0%']	
98	ER positive, chemotherapy decisions	A 57 year old has undergone breast conserving surgery for a stage 1, node-negative breast cancer, that is grade 2, ER positive, PR positive, and HER2 negative. She had tumor genomic testing, which shows a high-risk tumor. You would recommend adding chemotherapy to endocrine therapy for a tumor that was which size?	['a) I would give chemotherapy regardless of T size', 'b) 1.5cm', 'c) 1.2cm', 'd) 1.0cm', 'e) 0.8cm', 'f) 0.6cm', 'g) I would never give chemotherapy for a stage 1 tumor like this', 'h) Abstain']	['4.08%', '18.37%', '8.16%', '28.57%', '0.0%', '8.16%', '22.45%', '10.2%']	['100.0%', '0.0%', '0.0%', '0.0%', '0.0%', '0.0%', '0.0%', '0.0%']	-0.350
99	ER positive, chemotherapy decisions	Patients with invasive lobular breast cancer (grade 1 or 2, strongly ER positive, HER2 negative, stage 1-3, and without pleomorphic features) should not receive neo-/adjuvant chemotherapy.	['a) True', 'b) False', 'c) Abstain']	['36.0%', '60.0%', '4.0%']	['100.0%', '0.0%', '0.0%']	0.082
100	ER positive, chemotherapy decisions	Patients with classical invasive lobular breast cancer (grade 1 or 2, strongly ER and PR positive, Ki67 <10%, HER2 negative, and without pleomorphic features), and confirmation of favorable pathology by means of a low genomic signature score, should not receive neo- oder adjuvant chemotherapy, if stage 1,2 or 3.	['a) True', 'b) False', 'c) Abstain']	['63.46%', '30.77%', '5.77%']	['100.0%', '0.0%', '0.0%']	0.902
101	ER positive, chemotherapy decisions	A 38 year old patient is receiving GnRH agonist and AI therapy for stage 2 breast cancer. She is amenorrheic and experiencing menopausal symptoms. Her monitoring for funtctional menopause should include:	['a) No additional testing', 'b) Annual monitoring of estradiol levels', 'c) Semi-annual monitoring of estradiol levels', 'd) Abstain']	['36.54%', '5.77%', '44.23%', '13.46%']	['0.0%', '100.0%', '0.0%', '0.0%']	-0.700
102	ER positive, chemotherapy decisions	A 39 year old patient is receiving GnRH agonist and AI therapy for stage 2, ER positive, HER2 negative breast cancer. She has breakthrough menstrual bleeding on every 3 month GnRH agonist therapy. You would recommend:	['a) Monthly GnRH agonist', 'b) Switch to tamoxifen', 'c) Oophorectomy and continue AI', 'd) Abstain']	['64.62%', '12.31%', '6.15%', '16.92%']	['100.0%', '0.0%', '0.0%', '0.0%']	0.986
103	ER positive, chemotherapy decisions	A premenopausal woman in her 40s with stage 3 breast cancer is concluding 5 years of GnRH agonist therapy and paired with a non-steroidal aromatase inhibitor. She has had substantial arthralgias but otherwise found treatment to be acceptable. As ongoing therapy, you would recommend:	['a) Discontinue treatment', 'b) Tamoxifen', 'c) GnRH and tamoxifen', 'd) GnRH and same AI', 'e) GnRH and exemestane', 'f) Abstain']	['7.69%', '35.38%', '7.69%', '23.08%', '4.62%', '21.54%']	['0.0%', '0.0%', '0.0%', '0.0%', '100.0%', '0.0%']	-0.491
104	Triple Negative Therapy	Carboplatin should be included in the chemotherapy regimen for patients receiving neoadjuvant therapy for stage 2 or 3 TNBC who are also receiving taxane, anthracycline, and cyclophosphamide based chemotherapy when pembrolizumab is being administered.	['a) Yes', 'b) No', 'c) Abstain']	['78.0%', '12.0%', '10.0%']	['100.0%', '0.0%', '0.0%']	1.000
105	Triple Negative Therapy	Carboplatin should be included in the chemotherapy regimen for patients receiving neoadjuvant therapy for stage 2 or 3 TNBC who are also receiving taxane, anthracycline, and cyclophosphamide based chemotherapy when pembrolizumab is not being administered.	['a) Yes', 'b) No', 'c) Abstain']	['72.0%', '16.0%', '12.0%']	['100.0%', '0.0%', '0.0%']	0.998
106	Triple Negative Therapy	Multiple studies have suggested that dose-dense adjuvant chemotherapy improves outcomes. Fort hat reason, the „AC/EC“ phase of the KN522 regimen with concurrent pembrolizumab should be given every 2 weeks, and not every 3 weeks as was done in the clinical trial.	['a) Yes', 'b) No', 'c) Unsure as safety and efficacy confirmation needed before abandoning 3- weekly option', 'd) Abstain']	['29.23%', '13.85%', '38.46%', '18.46%']	['0.0%', '0.0%', '100.0%', '0.0%']	0.812
107	Triple Negative Therapy	A healthy premenopausal woman has received taxane/carboplatin followed by AC chemotherapy, with concurrent pembrolizumab, as	['a) Yes', 'b) No', 'c) Abstain']	['58.49%', '32.08%', '9.43%']	['100.0%', '0.0%', '0.0%']	0.887

		neoadjuvant treatment for TNBC. If she has a pCR should she also receive adjuvant pembrolizumab?				
108	Triple Negative Therapy	A healthy 60 year old woman has a clinical T2N0 triple negative breast cancer, of about 2-3 cm in size. She is a candidate for BCS without needing neoadjuvant therapy. Your preferred approach would be:	['a) Neoadjuvant chemotherapy and pembrolizumab', 'b) Neoadjuvant chemotherapy', 'c) Surgery', 'd) Abstain']	['64.62%', '21.54%', '7.69%', '6.15%']	['0.0%', '0.0%', '100.0%', '0.0%']	-0.423
109	Triple Negative Therapy	Should we use neoadjuvant pembrolizumab-based chemotherapy for stage 1 TNBC?	['a) Yes', 'b) No, „chemotherapy only“ is appropriate neoadjuvant treatment for small\nTNBC tumors', 'c) No, I recommend primary surgery for small TNBC that do not clinically have indication for neoadjuvant treatment, and then make chemotherapy recommendations', 'd) Abstain']	['4.62%', '46.15%', '41.54%', '7.69%']	['0.0%', '0.0%', '100.0%', '0.0%']	0.504
110	Triple Negative Therapy	A 45 year old woman has undergone primary surgery for TNBC, disclosing a stage 2 cancer with positive nodal involvement. In addition to standard chemotherapy, should she receive adjuvant pembrolizumab?	['a) Yes', 'b) No', 'c) Abstain']	['30.0%', '62.0%', '8.0%']	['100.0%', '0.0%', '0.0%']	-0.106
111	Her2 Positive	For a patient with stage 1, HER2 positive breast cancer, the preferred adjuvant treatment is:	['a) TCHP (Docetaxel, carboplatin, trastuzumab, pertuzumab)', 'b) TH (Paclitaxel, trastuzumab)', 'c) TDM1 (trastuzumab emtansine)', 'd) Abstain']	['4.62%', '84.62%', '0.0%', '10.77%']	['60.0%', '40.0%', '0.0%', '0.0%']	0.323
112	Her2 Positive	For patients who present with clinically node-negative breast cancer, and who receive neoadjuvant TCHP, and achieve a pCR, the appropriate adjuvant regimen is:	['a) Trastuzumab', 'b) Trastuzumab and pertuzumab', 'c) Abstain']	['63.27%', '32.65%', '4.08%']	['0.0%', '100.0%', '0.0%']	-0.020
113	Her2 Positive	A patient with HER2 positive breast cancer receives neoadjuvant TCHP. At surgery there is residual disease that is HER2 negative by FISH and by IHC. Her adjuvant therapy should be:	['a) Trastuzumab emtansine', 'b) Anthracycline chemotherapy', 'c) Both', 'd) Abstain']	['56.92%', '15.38%', '9.23%', '18.46%']	['0.0%', '100.0%', '0.0%', '0.0%']	-0.297
114	BRCA Associated	A 43 year old patient has been diagnosed with stage 2, node-positive TNBC. She is also found to have a BRCA1 mutation. She receives neoadjuvant KN522. At surgery, she has resiudal disease. In the adjuvant setting, in addition to pembrolizumab, she should receive:	['a) Capecitabine', 'b) Olaparib', 'c) Each, given in sequence', 'd) Abstain']	['2.0%', '62.0%', '24.0%', '12.0%']	['0.0%', '0.0%', '100.0%', '0.0%']	-0.025
115	BRCA Associated	A 43 year old patient has been diagnosed with stage 3, node-positive ER positive HER2 negative breast cancer. She is also found to have a BRCA2 mutation. She receives neo-/adjuvant dose-dense ACT/T. In the adjuvant setting, in addition to optimal endocrine therapy, she should receive:	['a) Olaparib', 'b) Abemaciclib', 'c) Each, given in sequence', 'd) Abstain']	['37.25%', '5.88%', '49.02%', '7.84%']	['100.0%', '0.0%', '0.0%', '0.0%']	0.380
116	BRCA Associated	In addition to other standard treatments, all patients with BRCA1 or BRCA2 mutations who receive neo-/adjuvant chemotherapy should receive platinum-based treatment.	['a) True', 'b) False', 'c) Abstain']	['37.0%', '50.0%', '12.0%']	['100.0%', '0.0%', '0.0%']	0.179
117	Bone Modifying Therapy	Adjuvant bone modifying therapy should be recommended to which group of woimen with postmenopausal breast cancer?	['a) All', 'b) All with ER positive tumors but not ER negative tumors', 'c) Only those with stage 2 or 3 (ER positive or ER negative)', 'd) Only those with stage 2 or 3, ER positive	['14.0%', '12.0%', '14.0%', '32.0%', '8.0%', '20.0%']	['0.0%', '60.0%', '40.0%', '0.0%',	-0.344

			only', 'e) Only those with stage 3 (ER positive or ER negative)', 'f) Abstain']		'0.0%', '0.0%']	
118	Oligometastatic Disease	A patient has been diagnosed with ER negative HER2 positive breast cancer, and staging scans disclose a 4cm tumor in the breast, positive axillary LN, and an isolated pulmonary nodule. The patient receives induction therapy with THP and has a complete clinical response. What is the recommended further therapy (question added by authors of this paper)?	['a) Yes, surgery alone.', 'b) Yes, radiation therapy alone.', 'c) Yes, both.', 'd) Neither', 'e) Abstain']	['10.0%', '8.0%', '68.0%', '14.0%', '0.0%']	['0.0%', '0.0%', '100.0%', '0.0%', '0.0%']	0.982
119	Oligometastatic Disease	A patient has been diagnosed with ER negative HER2 negative breast cancer, and staging scans disclose a 4cm tumor in the breast, positive axillary LN, and an isolated pulmonary nodule. The patient receives induction therapy with taxane/carboplatin and has a complete clinical response. What is the recommended further therapy (question added by authors of this paper)?	['a) Yes, surgery alone.', 'b) Yes, radiation therapy alone.', 'c) Yes, both.', 'd) Neither', 'e) Abstain.']	['7.69%', '4.62%', '63.08%', '20.0%', '4.62%']	['0.0%', '0.0%', '100.0%', '0.0%', '0.0%']	0.967
120	Oligometastatic Disease	A patient has been diagnosed with ER positive HER2 positive breast cancer, and staging scans disclose a 4cm tumor in the breast, positive axillary LN, and an isolated pulmonary nodule. The patient receives induction therapy with CDK4/6i and AI treatment, and has a complete clinical response. What is the recommended further therapy (question added by authors of this paper)?	['a) Yes, surgery alone.', 'b) Yes, radiation therapy alone.', 'c) Yes, both.', 'd) Neither', 'e) Abstain.']	['7.69%', '3.08%', '56.92%', '21.54%', '10.77%']	['0.0%', '0.0%', '100.0%', '0.0%', '0.0%']	0.950
121	Oligometastatic Disease	A patient has stage 2 breast cancer on one side and is found to have an isolated contralateral axillary LN. This patient should receive definite therapy with curative intent including contralateral axillary surgery and radiation therapy, and adjuvant treatment as standard.	['a) Yes', 'b) No', 'c) Abstain']	['75.0%', '15.63%', '9.38%']	['100.0%', '0.0%', '0.0%']	0.996
122	Molecular Diagnostics	Patients with early stage breast cancer should have ctDNA testing after surgery to determine their risk for future recurrence	['a) Yes', 'b) No', 'c) Abstain']	['14.0%', '86.0%', '0.0%']	['20.0%', '80.0%', '0.0%']	0.996
123	Molecular Diagnostics	Routine use of ctDNA in early breast cancer should happen when there is	['a) Adequate assembly of data proving prognostic significance', 'b) Only after prospective studies have shown that testing has therapeutic implications', 'c) Abstain']	['10.77%', '89.23%', '0.0%']	['0.0%', '100.0%', '0.0%']	0.994
124	Molecular Diagnostics	A patient has received neoadjuvant therapy a spart of a clinical trial. At the end oft he trial she undergoes breast surgery, an das part oft he trial, has a sample sent to test for presence of ctDNA. Should this result be shared with clinician?	['a) Yes', 'b) No', 'c) Abstain']	['0.4462%', '49.23%', '6.15%']	['100.0%', '0.0%', '0.0%']	-0.590
125	Molecular Diagnostics	A patient has received neoadjuvant therapy as part of a clinical trial. At the end oft he trial she undergoes breast surgery, an das part of the trial, has a sample sent to test for presence of ctDNA. Should this result be shared with the patient?	['a) Yes', 'b) No', 'c) Abstain']	['44.62%', '50.77%', '4.62%']	['100.0%', '0.0%', '0.0%']	0.390
126	Molecular Diagnostics	A patient has received neoadjuvant chemotherapy/trastuzumab/pertuzumab as part of clinical trial. At the end oft he trial she undergoes breast surgery, with a pCR. As part of the trial, a test sent for presence of ctDNA shows positive residual tumor DNA.Should she receive trastuzumab-emtansine?	['a) Yes', 'b) No', 'c) Abstain']	['0.1538%', '69.23%', '15.38%']	['100.0%', '0.0%', '0.0%']	-0.671

127	Molecular Diagnostics	Five years ago, a postmenopausal woman received standard therapy for a stage 3, ER positive, HER2 negative breast cancer. She is eligible for a clinical trial in which ctDNA testing is performed to determine ongoing treatment. In the trial, patients whose ctDNA discloses an ESR1 mutation are randomized to ongoing aromatase inhibitor therapy or a switch to fulvestrant. In light of data from the metastatic setting, are you at equipoise that this is a fail study?	['a) Yes', 'b) No', 'c) Abstain']	['43.08%', '38.46%', '18.46%']	['0.0%', '100.0%', '0.0%']	0.339
